# Being touched by death while giving birth to life: A meta-ethnography exploring women’s experiences with postpartum hemorrhage

**DOI:** 10.18332/ejm/200615

**Published:** 2025-03-26

**Authors:** Kristine E. Allum, Synne Tilly, Bente Dahl

**Affiliations:** 1 Centre for Women’s, Family and Child Health, Faculty of Health and Social Sciences, University of South-Eastern Norway, Borre, Norway

**Keywords:** qualitative, postpartum haemorrhage, traumatic birth, meta-ethnography, existential experience

## Abstract

**INTRODUCTION:**

Postpartum hemorrhage (PPH) is a serious complication following childbirth and the most common cause of maternal mortality globally. Women who survive PPH have an increased risk of enduring long-term physical and psychological consequences. This meta-ethnography aimed to investigate women’s experiences of postpartum hemorrhage to develop new insights and understanding of women's needs for care and follow-up after a PPH.

**METHODS:**

A meta-ethnography was carried out in accordance with Noblit and Hare’s seven phases and the eMERGe reporting guidance. Comprehensive literature searches were conducted in MEDLINE, CINAHL, and Maternity and Infant Care, in January 2024. A PRISMA flowchart was used to illustrate the search process and quality assessment was performed according to CASP.

**RESULTS:**

Eight studies were included based on specific inclusion criteria. The analysis and synthesis led to the overarching metaphor of ‘Being touched by death while giving birth to life’ encompassing three main themes. The first theme, ‘When death roams by’ described women’s experiences with PPH as an encounter with death, leading to fear and severe pain. The second theme ‘Living on with an emotional scar’ indicated how PPH was an experience leaving deep impact in a person. The final theme, ‘Healthcare providers as anchors amid the chaos’, described that women valued healthcare professionals who demonstrated empathy and good communication skills.

**CONCLUSIONS:**

Our study emphasizes the importance of healthcare providers, particularly midwives, providing empathetic care to aid women in recovering from postpartum hemorrhage.

## INTRODUCTION

Postpartum hemorrhage (PPH) is a serious complication of childbirth. According to the World Health Organization (WHO), PPH can be defined as ‘blood loss of 500 mL or more within 24 hours after birth, whereas severe PPH is defined as blood loss of 1000 mL or more within the same timeframe’^1^. The most common cause of PPH is uterine atony, but PPH can also be caused by trauma, placental and membrane remnants, or coagulation disorders. Although there are several known risk factors, most cases of PPH occur completely unexpectedly^1^.

Postpartum hemorrhage affects millions of women every year and results in more than 70000 maternal deaths annually, most of which are in low- and middle-income countries. This makes PPH the most common cause of maternal mortality worldwide, although most deaths related to PPH could have been avoided with proper prevention and treatment^1-3^.

Several studies have demonstrated an increased prevalence of PPH among several high-income countries^4-7^. A recent study noted that the reason for the increase is not entirely clear but refers to increased risk factors in women who are giving birth today, such as high maternal age, high bodi mass index (BMI), and previous cesarean sections. In addition, there are increased interventions in obstetric care, such as induction and vaginal operative deliveries^8^. Women who survive PPH have an increased risk of being left with a traumatic birth experience^9^, and several international studies, including a report from the World Health Organization, point to a greater risk of depressive and physical symptoms after PPH^2,10,11^. A systematic review revealed a lack of studies describing the extent of physical and psychological consequences after a PPH and a need for further research^12^.

In 2023, the WHO launched a new roadmap to reduce preventable maternal deaths related to PPH by 2030. The roadmap contains priority actions and a timeline. The roadmap is also a joint plan to prevent millions of women from experiencing long-term consequences as a result of a traumatic birth experience^2^. The WHO emphasizes the importance of looking beyond the survival of PPH and points to increased global attention to individualized care that focuses on women^13^. The shift in focus results from quantitative studies conducted over a long period of time, the purpose of which has been to calculate outcomes and effects. However, this method fails to consider all aspects of what truly matters to women. To balance the evidence, more qualitative studies are needed that examine women’s perceptions and experiences of PPH^14^.

The objective of this meta-ethnography is to explore women’s experiences of PPH to develop new insights and understanding of women’s need for care and follow-up after a PPH.

To address the ‘knowledge gap’ we found a meta-ethnographic approach15 to be an appropriate tool. This approach would allow healthcare professionals, particularly midwives, to improve care during this critical period by synthesizing a broader exploration of women’s perceptions, experiences, and emotions.

## METHODS

This study used meta-ethnography, an interpretative methodology for qualitative evidence synthesis. Meta-ethnography aims to analyze and synthesize rather than aggregate findings from qualitative primary studies and has the potential to generate new knowledge, insights, and theory^15-17^. The process of synthesizing findings is performed by ‘translating qualitative studies into one another’^15^, which means that findings from the included studies are identified, extracted, and compared with respect to similarities and differences. We conducted the study according to the seven phases of meta-ethnography described by Noblit and Hare^15^, as presented in Table 1. We adhered to the eMERGe meta-ethnography reporting guidance to ensure transparency and accuracy. The guidance is presented in Supplementary file Table 1 and consists of 19 different reporting criteria covering all phases of meta-ethnography^17^.

**Table 1:** Phases in meta-ethnography^15^

*Phases*	*Strategies*
1. Getting started	Identifying the topic of the study and defining the aim.
2. Deciding what is relevant to the initial interest	Including relevant studies, describing search strategy and criteria for inclusion and exclusion.
3. Reading the studies	Noting studies’ interpretative metaphors through repeated readings.
4. Determining how the studies are related	Determining the relationship between the studies by creating a list of key metaphors (themes, concepts, phrases, ideas) and assessing whether the relationships are reciprocal (i.e. findings across studies are comparable), refutational (findings stand in opposition to each other) or represent a line of argument.
5. Translating the studies into one another	Comparing metaphors and their interactions within single studies and across studies, and at the same time protecting uniqueness and holism.
6. Synthesizing translations	Creating a new whole from the sum of the part, enabling a second-level analysis.
7. Expressing the synthesis	Finding the appropriate form for the synthesis to be effectively communicated to the audience.

### Phase 1: Getting started

The first phase involved identifying the research objectives that could be addressed through a qualitative evidence synthesis. Our interest in this topic originates from our clinical maternity care practice. We noticed that many women experience PPH, but at the same time, we often found postpartum care and follow-up to be haphazard and inadequate. We noticed how the PPH affected women in retrospect and asked whether the follow-up was satisfactory. When conducting pilot searches in the MEDLINE, CINAHL, Maternity and Infant Care (MIDIRS), and PsycINFO databases from October 2023 to January 2024, we noticed that most research regarding PPH was quantitative. We were able to locate some qualitative studies describing women’s experiences of PPH, but we were unable to find literature reviews or syntheses that examined this.

### Phase 2: Deciding what is relevant to the initial interest

Phase two gives a detailed account of the process used to include relevant studies, including the search strategy and search process, the selection of studies, and the quality assessment.

*Search strategy and search processes*

The search strategy was guided by the research aim and the objective of the meta-ethnography to capture qualitative research focused on women’s perceptions and experiences of PPH. After adjusting the search terms used in the pilot searches, two authors (ST, KEA) conducted comprehensive literature searches in three different databases, MEDLINE, CINAHL, and MIDIRS, in mid January 2024 (Supplementary file Table 2). The search strategy and choice of search terms were developed in collaboration with a librarian. A PICO form adapted to qualitative studies was used (Supplementary file Table 3). No search terms were entered under ‘comparison’, as this limited the number of hits, and the purpose of the study was not to compare or calculate but to interpret and synthesize knowledge about women’s experiences of PPH^18,19^. The following search terms were employed: postpartum hemorrhage (keyword), postpartum hemorrhage*, pph*, postpartum hemorrhage*, postpartum bleeding*, women (keyword), women*, woman*, and experience*. Boolean operators were used to combine the terms, and truncations were used to ensure a broad search. No filters or delimitations were used. As MIDIRS does not have relevant keywords, this database was only searched for text words.

*Primary studies selection*

In the second phase of the meta-ethnography, we developed inclusion and exclusion criteria based on the pilot search and previous research published on the topic^15^. Qualitative peer-reviewed primary articles published in international journals describing women’s experiences with postpartum hemorrhage during and after birth were included. We chose not to use any delimitation but included studies describing women’s experiences, regardless of time. Articles including women with a PPH above 500 mL were included. No limitation was made with regard to the woman’s age, parity, or method of delivery. Articles written in English or one of the Nordic languages were included, and articles in other languages were excluded. Articles with different perspectives were included, where it was possible to distinguish the woman’s voice. Mixed-method articles with a qualitative component in which the woman’s voice was clear were included. No restrictions were made regarding the year of publication, as few articles had been published on the topic, and we did not want to risk losing good studies. Quantitative articles, Master’s theses, PhD theses, book chapters, and feature articles were excluded. Duplicates were removed before the articles were screened by reading headlines and abstracts. Relevant articles were considered for inclusion. Backtracking of the reference lists was performed, resulting in one included article.

**Figure 1:**
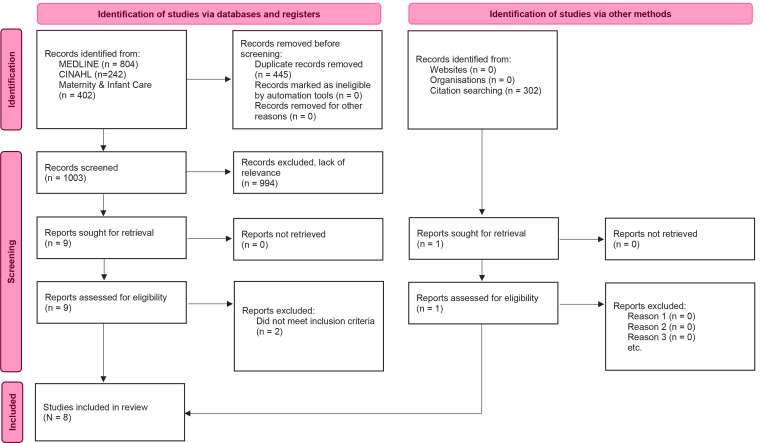
The PRISMA 2020 statement: an updated guideline for reporting systematic reviews^20^

The study selection process consisted of three steps and was conducted by two authors (ST, KEA). We started by removing duplicates and screened titles to remove irrelevant records. In the following step, abstracts were read and assessed for eligibility. Nine articles were read in full text and assessed for relevance according to the inclusion and exclusion criteria. This resulted in the exclusion of two articles. The manual search yielded one additional article. A total of eight articles were included in the final review. The literature search and screening process is described in detail in a PRISMA flow diagram^20^ (Figure 1).

*Quality assessment*

All the articles were assessed for quality via a checklist for qualitative studies from the Critical Appraisal Skills Program (CASP), including the study’s purpose, method, recruitment method, data collection, reflexivity, ethical considerations, data analysis, results, and implications for practice^21^. The quality assessment was carried out in collaboration with two authors (ST, KEA) and is presented in Table 2. Two of the studies used a mixed method; these studies were also assessed, but we considered only the qualitative part of the studies. Two studies did not account for reflexivity but answered the remaining questions well and were included after an overall quality assessment. The remaining studies answered all the questions in the CASP form well and were included.

**Table 2:** Quality assessment of included studies (CASP)^21^

*Authors*	*Year*	Assessment questions*									
		*1*	*2*	*3*	*4*	*5*	*6*	*7*	*8*	*9*	*10*
Briley et al.^26^	2021	Y	Y	Y	Y	Y	Y	Y	Y	Y	Y
de la Cruz et al.^25^	2013	Y	Y	Y	Y	Y	Y	Y	Y	Y	Y
Dunning et al.^23^	2016	Y	Y	Y	Y	Y	Y	Y	Y	Y	Y
Elmir et al.^24^	2012	Y	Y	Y	Y	Y	Y	Y	Y	Y	Y
Robertson et al.^29^	2017	Y	Y	Y	Y	Y	N	Y	Y	Y	Y
Rosvig et al.^22^	2023	Y	Y	Y	Y	Y	Y	Y	Y	Y	Y
Snowdon et al.^27^	2012	Y	Y	Y	Y	Y	Y	Y	Y	Y	Y
Thompson et al.^28^	2021	Y	Y	Y	Y	Y	C	Y	Y	Y	Y

Y: yes. N: no. C: cannot tell.

* Assessment questions: 1) Was there a clear statement of the aims of the research?; 2) Is a qualitative methodology appropriate?; 3) Was the research design appropriate to address the aims of the research?; 4) Was the recruitment strategy appropriate to the aims of the research?; 5) Were the data collected in a way that addressed the research issue?; 6) Has the relationship between researcher and participants been adequately considered?; 7) Have ethical issues been taken into consideration?; 8) Was the data analysis sufficiently rigorous?; 9) Is there a clear statement of findings?; and 10) How valuable is the research?

*Ethical considerations*

We used data from peer-reviewed primary research articles in the meta-ethnography. Therefore, there was no need for ethical approval. Nevertheless, we ensured that all the primary articles had attained ethical approval before the commencement of their respective studies.

### Phase 3: Reading the studies

In the third phase, two authors read the studies several times (ST, KEA) to achieve critical reading.

*Data extraction approach*

The repeated reading provided an overview of the data material and a preliminary identification of various metaphors. Data extraction was performed. The results sections of the primary studies were read line-by-line by two authors (ST, KEA), and metaphors that could provide insight into the study’s objectives were processed into a matrix. The metaphors encompassed first-order concepts (direct quotes from participants) and second-order concepts (interpretations offered by the authors, presented in the form of concepts, themes, and categories). A primary study with rich data material and acceptable methodological quality was selected as an index study^22^. The index study was placed in the leftmost column in the matrix, and metaphors from the study were placed in a column. The same was done with the remaining seven studies. The literature matrix is presented in Supplementary file Table 4. Information on the included studies describing the study aim, sample characteristics, research design, data collection, data analysis, and key findings, is presented in the Results section (Table 3).

**Table 3:** Characteristics of included studies

*Authors Year Country*	*Aim*	*Sample and setting*	*Methods for data collection and analysis*	*Key findings*
Briley et al.^26^ 2021 England	To qualitatively investigate the experience of PPH, for women, birth partners, and HCPs in an inner city tertiary referral center. To provide multi-faceted insight into PPH and improve understanding and future care practices.	Postnatal wards in two South London NHS Trusts Five women, four women and birth partner dyads, and nine HCPs involved in the management of the PPHs (two consultant obstetricians; two research midwives; three midwives; and two student midwives) (n=22)	Semi-structured interviews Thematic analysis (Braun and Clarke)	Four distinct, but related, themes were identified: ‘Knowledge specific to PPH’; ‘Effective and appropriate responses to PPH’; ‘Communication of risk factors’; and ‘Quantifying blood loss’; which collected around a central organizing concept of ‘Explaining the indescribable’.
de la Cruz et al.^25^ 2013 USA	To explore women’s experiences of emergency peripartum hysterectomy to make recommendations for care.	English speaking international Internet support group of EPH survivors. 15 women living in the US, UK and Canada	Individual telephone interviews Constant Comparative Analysis/Grounded theory.	Seven major themes were identified: fear; pain; death and dying; numbness or delay in emotional reaction; bonding with baby; communication; and the need for information. A major finding is the need for additional follow-up visits to address the emotional after-effects and to fill in gaps in women’s understanding and memory of what had occurred.
Dunning et al.^23^ 2016 England	To investigate the experiences of women who have had a primary PPH of varying volumes (major, moderate and minor), and the experiences of birth partners who witnessed the PPH.	London teaching hospital 11 women and 6 partners (n=17)	Semi-structured interviews Matrix-based thematic method	Four main themes emerged from the analysis: control, communication, consequence and competence. Many were unaware they had a PPH, and would have preferred more information either at the time or in the postnatal period. Birth partners also required more information, especially if separated from their partner during the PPH.
Elmir et al.^24^ 2012 Australia	To describe women’s experiences of having an emergency hysterectomy following a severe postpartum hemorrhage.	Three states in Australia: New South Wales, Victoria and Western Australia 21 Australian women	Semi-structured, face to face, telephone and internet email interviews Inductive analysis (Lincoln and Guba)	A major theme, ‘between life and death’ and three sub-themes were identified, ‘being close to death: bleeding and fear’; ‘having a hysterectomy: devastation and realization’; and ‘reliving the trauma: flashbacks and memories’.
Robertson et al.^29^ 2017 Canada	To describe the experiences of midwifery clients in Ontario who had suffered a PPH and to compare those findings to what is documented in existing literature.	Former Ontario midwifery clients having given birth at home or in hospital, being part of the AOM’s consumer involvement program in the development of the PPH clinical practice guideline. 23 women	Two semi-structured focus group interviews and two online surveys Thematic analysis	A range of physical and emotional responses to the experience of PPH were identified, ranging from no effect to short- or long-term psychological trauma which is consistent with a small but growing body of international studies.
Rosvig et al.^22^ 2023 Denmark	To examine women and their partners’ experience of major PPH.	One regional hospital and one university hospital in Denmark 6 women and 9 couples (n=15)	Semi-structured interviews via video conference Thematic analysis (Braun and Clarke).	Three major themes were identified: 1) ‘From birth to emergency’, 2) ‘Feeling safe during an emergency’, and 3) ‘Family unity challenged’.
Snowdon et al.^27^ 2012 England	To explore how severe PPH and its management is experienced by women and their partners, and how they later view events.	Participants’ homes, focusing on experiences of PPH in hospital and post-discharge 9 women and 6 partners (n=15)	Individual and joint semi-structured interviews Thematic analysis	A dominant theme of communication difficulties, and two subthemes, disempowerment and information-deprivation were identified. Communication difficulties were understandable during the emergency but were frustrating and upsetting in postpartum care and the longer term.
Thompson et al.^28^ 2011 Australia New Zealand	To explore women’s experiences of care and their concerns and needs after a significant primary PPH.	17 major women’s and general hospitals with maternity services 206 women	Questionnaire with open-ended question Thematic analysis	Four major themes were identified in response to the open-ended questions: adequacy of care, emotional responses to the experience, implications for the future, and concerns for their baby.

PPH: postpartum hemorrhage. HCP: healthcare providers. EPH: emergency peripartum hysterectomy.

**Table 4:** Themes, subthemes and their references

*Themes*	*Subthemes*	*References*
When death roams by	The fear of dying Stronger pain than birth pain Disconnecting from the body to endure an unbearable situation	22–25, 27–29 22, 24, 25, 27–29 22–24
Living on with an ‘emotional scar’	Feelings of loss, disappointment and guilt over an experience that did not turn out as it should have Finding meaning to be able to move forwards	22–25, 28, 29 22–25, 27–29
Healthcare providers being ‘anchors’ amid the chaos	The importance of being recognised as a ‘real’ person A desire to understand	22, 23, 25, 26, 28 22, 23, 25–28

### Phase 4: Determining how the studies are related

Phases 4 to 7 include the analysis and synthesis process^15^. This was carried out in collaboration with all the authors. Consistent with the meta-ethnographic approach^15^, all relevant interpretative metaphors identified in the results sections of the included studies were considered empirical material for the meta-ethnography. The matrix was a tool for organizing and comparing metaphors across the studies. The selected metaphors were assessed in relation to each other, focusing on how they were related^15^. The studies can be potentially comparable, allowing for a reciprocal translation to be conducted. In cases where the studies contradict each other, a refutational synthesis is conducted. If the studies illuminate different aspects of the subject, a lines-of-argument synthesis can be carried out^15^. We started with an assumption that the findings were primarily reciprocal. However, we also considered whether the studies covered different aspects of the phenomenon.

### Phase 5: Translating the studies into one another

Phase 5 evaluates the initial assumptions about the relationship between the studies’ findings as they appeared in phase 4. The translation process is a crucial aspect of meta-ethnography^15^. In this study, we employed analog translation to interpret the findings. Our understanding emerged as a translation of the metaphors into a new expression (third-order concept)^15^. This process was inductive and iterative and involved moving back and forth in the data. All authors participated in the process. The initial draft was prepared by two authors (ST, KEA). Following this, the research group reviewed and discussed the draft before we arranged the translations into sub-themes and themes. The translation process is described in Supplementary file Table 5.

### Phase 6: Synthesizing translations

The interpretative process identified various aspects of our topic that moved beyond the original primary studies. During the process, arguments were synthesized; our analysis perspective became a lines-of-argument synthesis^15^. In this interpretative process, a new understanding or storyline emerged in the dissemination of the translations. All authors were involved in the process, and we assessed and discussed our interpretations before reaching a consensus. The analysis and synthesis resulted in the three main themes given in Table 4.

### Phase 7: Expressing the synthesis

The overarching lines-of-argument synthesis ‘Being touched by death while giving birth to life’ encompassed all the main themes and subthemes. The synthesis, conveyed in a text, consists of descriptive data and includes quotes from the primary studies. It should interest healthcare providers (HCP) and other interested parties.

## RESULTS

We screened 1008 articles for relevance. Due to unsuitable methodologies and perspectives, lack of relevance, and inadequate findings, most articles were excluded. However, nine articles were examined in full text, and eight articles were included: seven articles from the literature search and 1 article located through backtracking from the articles’ reference lists. Regarding the quality assessment of the included articles, two answered 9 out of 10 questions on the CASP checklist, while the rest answered all 10. Consequently, all studies were considered to be of sufficiently high quality to be included in the meta-ethnography.

The meta-ethnography included eight articles published between 2011 and 2023. The studies were conducted in 6 different countries: Australia, New Zealand, England, USA, Canada and Denmark. Three hundred nine women were included, with samples ranging from 9 to 206 women. Three studies examined women’s and partners’ experiences of PPH, four examined women’s experiences only, and 1 study examined women’s partners’ and healthcare providers’ (HCP) experiences. Two studies also described women’s experiences of acute hysterectomy; these data have not been used in the meta-ethnography. Six studies used semi-structured interviews^22-27^. One study used a questionnaire asking: ‘Is there anything about your labor and birth that is bothering you now?’, where the woman could write down her answer. The questionnaires were sent to the women at one week, two months, and four months postpartum^28^. Another study used questionnaires with open-ended questions and focus group interviews with identical interview guides. The questions included the women’s experiences of postpartum hemorrhage, emotional and physical consequences, and how the postpartum hemorrhage affected family members. In addition, the women were asked to reflect on the care they received^29^. Seven studies analyzed the data material via different methods of thematic analysis^22-24,26-29^. One of the studies used grounded theory^25^.

### Synthesis

The analysis of the included studies led to the overarching metaphor: ‘Being touched by death while giving birth to life’. The metaphor was identified during the analysis when the contrasts in the women’s experiences became visible. The women had expectations of a birth that would end with the joy of having a newborn baby. The contrast was, therefore, great when the birth instead ended with a PPH, and emotional chaos ensued. The theme ‘death roams by’ describes how women experienced that death suddenly came close. The fear of dying, combined with severe pain, made the situation unbearable. As a result, the women experienced a divide between their consciousness and the body – they saw themselves from the outside. The traumatic experience led to an ‘emotional scar’. The emotional scar did not disappear even though the PPH was managed and the birth ended with a healthy child. It became difficult for the women to move on without processing their experience. During the process, support from HCPs was essential and enabled the women to use their resources. Using their resources opened a door that allowed the women to come strengthened out of the experience. For these women, HCPs became ‘anchors’ amid emotional chaos.

**Figure 2:**
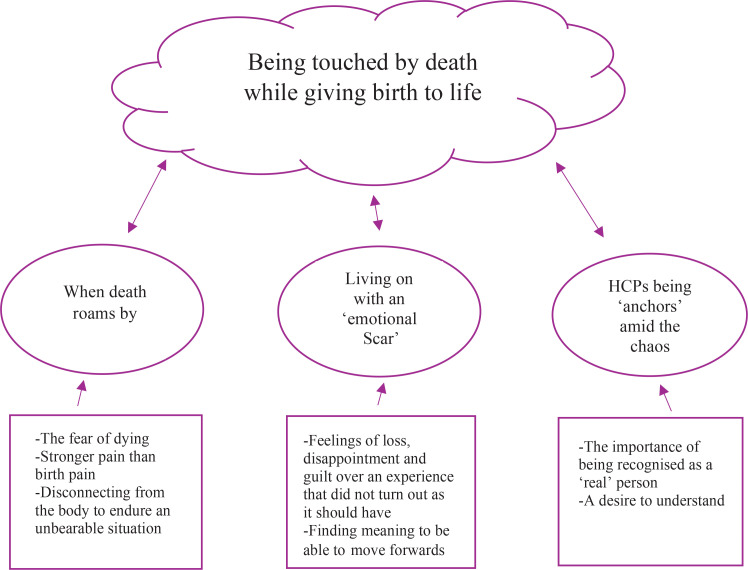
Model

The synthesis comprises three different themes: 1) When death roams by, 2) Living on with an ‘emotional scar’, and 3) Healthcare providers as ‘anchors’ amid the chaos. The themes comprise two to three subthemes and are presented in Figure 2 and Table 4.

### When death roams by

The women reported that postpartum hemorrhage caused their situation to change from being one of safety to suddenly becoming serious and potentially life-threatening^22,24,25,27,28^. The first topic that emerged in our analysis describes this serious situation, which the women experienced as an encounter with death, leading to fear, severe pain, and the feeling of being ‘disconnected’ from their bodies.

*The fear of dying*

When the alarm sounded, and more HCPs were called into the birthing room, the women noticed that the atmosphere and communication between the HCPs had changed. At that moment, the severity of the situation became clear to them. The women described how they felt being between life and death. For some, the fear of dying was all-consuming, whereas others dismissed their thoughts as being irrelevant or avoided thinking about it^22,24,25,27^. The women described how they ‘felt’ that they were losing much blood, and they noticed that the physical reactions to the bleeding, such as feeling cold, intensified the feeling of being close to death^22,24,25^. One woman described it as follows:

*‘I*’*d lost so much blood so quickly, I was dying, basically, and I could feel myself dying and sort of slipping away which. I felt like, you know those really old TVs, where you turn them off and the screen sort of disappears into a little circle before it goes black.’* ^24^

In addition to the fear of dying, the women also had thoughts about how their death would affect the family and the newborn child. The women described how they envisioned the newborn child growing up without a mother and how their partner would handle being left alone with the child^24,25,27^. Thoughts about death and its consequences became something that followed the women afterward. Many struggled with flashbacks and nightmares about dying^24,25,27-29^. In addition, the experience led to a strong fear of losing their partner or child^24,28^. One woman expressed this as follows:

*‘I think that as a result of what felt like a ‘‘brush with death”, I am more anxious about losing my partner or baby. They are more precious than ever.’* ^28^

The women’s experiences of a lack of control reinforced their fear of dying. Some women had great confidence in the HCPs and felt confident in relinquishing control^22,23,29^. Others struggled with the feeling of not being in control and had a fundamental distrust of the HCPs where they were in doubt as to whether the management of the postpartum hemorrhage was good enough^24,27,28^. Several studies demonstrated how separation from their partner and child reinforces women’s fears. The delivery room was described as safe, and the transfer to the operating theatre was a stark contrast that confirmed the severity of the situation^22,24^.

*Stronger pain than birth pain*

The pain resulting from the postpartum hemorrhage was described as stronger than the birth pain. Several women had memories of shouting and screaming and trying to push HCPs hands away during the bleeding^22,25,27,29^. One woman described the pain as follows:

*‘I just remember lots of people pummeling at my stomach in shifts, like two people at a time, pummeling my stomach. And I can remember it hurting and just actually trying to push them away.’* ^24^

Manual procedures performed in the delivery room, such as aortic compression, uterine compression, and bimanual uterine compression, are traumatic due to the resulting extremely severe pain. The women described a desire just to be ‘disconnected’. The pain overwhelmed everything else^24,25,28^. In retrospect, some women described how they were unable to close their eyes without reliving the performance of manual procedures by HCPs that caused extreme pain^25^.

*Disconnecting from the body to endure an unbearable situation*

When the bleeding occurred and the situation became serious, some women described having an ‘out-of-body’ experience, where they felt that they could see themselves from the outside. They felt helpless, weak, and unable to do anything about the situation. They observed that the HCPs worked quickly around them^22-24^, and they lost their sense of time^23^. One woman expressed this as follows:

*‘I just think I surrendered myself completely to the situation. Like, I am completely powerless, and I have absolutely no clue what’s going on. So, I was just like … “do whatever you want with me”, you know. Because you are ... completely surrendered to the situation and I was ready for ... whatever works, right?’* ^22^

The acute situation was characterized by fear and pain, and the women described how they disconnected from their bodies as a way of staying calm^22-24^. For some women, having the newborn child with them helped, as the child became a ‘filter’ for the chaos surrounding them^22^. Several studies demonstrated that the experience of ‘seeing oneself from the outside’ deeply affected the women^23,24^.

### Living on with an ‘emotional scar’

The analysis of the included studies demonstrated how a postpartum hemorrhage is more than a ‘there and then’ phenomenon; it is an experience that leaves deep traces and ‘emotional scars’ in a person. The experience led to feelings of loss, disappointment, and guilt. The women described a great need to find meaning in the situation to be able to move forward with their lives^22-25,27-29^.

*Feelings of loss, disappointment, and guilt over an experience that did not turn out as it should have*

Several studies concluded that the women took a long time to recover physically after the PPH. Poor physical condition created feelings of disappointment and loss^23,24,28^. They compared themselves to healthy postnatal women and were disappointed that they were not in the same shape as these other new mothers^28^. The women described using iron supplements for several months afterward. They found that everyday activities such as going for a walk, taking a shower, and taking care of their child were difficult^23,28^.

It was only when their physical condition began to improve that the shock of the postpartum hemorrhage began to subside. However, they still experienced painful memories, nightmares, and fear for a long period of time^22,24,25,28,29^. Several of the women were hesitant to have a new pregnancy, and some made a choice not to have any more children. The fear of reliving a postpartum hemorrhage and the loss of the belief that they would be able to ‘safely’ give birth to a child outweighed any desire for a new child^22,28,29^. One woman said:

*‘I must admit that the thought of going through labor again scares the wits out of me. I almost feel like I cheated death once and don’t want to tempt fate.’* ^28^

The feeling of loss was also linked to separation from the newborn child^22-25,28,29^. The first encounter with the child was not as magical as the women had imagined^22^. For some, exhaustion was so all-consuming that they did not feel an immediate concern for their child. This led to feelings of guilt later on^25^. Their reduced physical fitness also meant that the women needed a lot of help, resulting in feelings of being inadequate^22,25,28^.

Breastfeeding had become a challenge for most of the women. Those who achieved partial or exclusive breastfeeding experienced this as ‘healing’. The women talked about how they had not ‘managed’ to give birth to their child safely but that they had at least managed to breastfeed. Feelings of loss, disappointment, and guilt were prominent in women who were unable to breastfeed^23,25,28,29^.

*Finding meaning to be able to move forwards*

Most of the women described how the postpartum hemorrhage affected them emotionally, and the ‘emotional scar’ was the greatest concern for the women afterwards^23,25,28,29^. They needed to process the experience and fill in ‘gaps’ in their memories to move on. Many experienced a lack of follow-up afterward and tried to find information about the incident themselves with the help of their partner, hospital records, or internet searches^23-25,27,28^.

Several studies showed that the women were aware of the kind of support they had expected and hoped for after the PPH. However, many found that they were unable to speak up if they did not receive the help they needed^22-25,27-29^. One woman described how not being able to stand up for herself affected her self-esteem and made her feel like a victim:

*‘[It] has affected the way that I think about myself, the way I conduct myself, my life everything, because I’ve never got over that anger with myself for putting up with what I put up with there. I was just a complete victim.’* ^27^

Many women struggled with triggers that caused them to relive the event. Examples of triggers could be smells, sounds, or the sight of healthcare personnel. Follow-up consultations and contact from healthcare personnel during examinations could also lead to reliving the memories. In addition, a lack of control became a trigger for some^24,25,29^. One woman put it this way:

*‘I still get very anxious. Ever since it happened I’ve hated being a passenger in a car and even now, I don’t know if it’s the loss of being out of control but I can barely be a passenger in a car. I always have to be the person driving, and I was never like that before and I just have this fear of car accidents.’* ^24^

### Healthcare providers being ‘anchors’ amid the chaos

Postpartum hemorrhage led to emotional chaos, and for some women, HCPs became ‘anchors’ amid the chaos. The women valued healthcare professionals who showed emotion and communicated in a caring and sincere manner22,23,25-28.

*The importance of being recognized as a ‘real’ person*

Many women stated that the HCPs’ quick and effective emergency handling made them feel safe^22,23,26^. In addition to medical security, it was important for women that HCPs recognized their emotional needs. Many of the women felt that they had been deprived of the ‘perfect birth’ and felt a sense of grief as a result. The women wanted to be treated with respect, sensitivity, and compassion. They wanted to feel that HCPs genuinely wanted to help them^23,25,26,28^. One woman described how a lack of recognition caused her to lose all her trust:

*‘I did not know the midwife before the delivery, and I found her to be very unsupportive and impatient towards me. When I began hemorrhaging shortly after delivery, she made a great effort not to acknowledge me as she tried to stop the bleeding. But as she failed to hide her panic and she had never once assured me that I could trust her, I was terrified!’* ^28^

The women valued healthcare professionals who provided genuine and personal care and took the time to reassure them^23,25^. The fact that HCPs showed emotion was a strong and powerful experience for the women. This sincerity resulted in feelings of true compassion, and the HCPs saw them as ‘real’ people^25^. One woman described it as follows:

*‘You always have to be professional but ... it’s OK to let your barriers down as a health care provider, it’s OK to say, “Oh my God, we were so scared”, “Oh my God, that was so awful ... let’s talk about it together”. That was a wonderful thing. That was my experience, which was very positive ... That was a very powerful thing, to feel like I was a real person to those people, and that they care too and that they hurt too (crying).’* ^25^

*A desire to understand*

Several of the studies indicated that women had a strong need for information after the PPH. The lack of information was described as traumatizing. The women wanted to understand what had happened and what would happen next. The way in which HCPs communicated and the timing of the conversation was just as important as the content of the conversation^23,25-27^. When decisions were made, several of the women felt that the information was given in an informative way, without leading to panic^22,25,26^. However, receiving information when they were not ready could increase feelings of fear^23^. Some women said that they felt it was inappropriate to receive information about the postpartum hemorrhage immediately after the event and that they were unable to understand it at the time^26^.

Poor staffing and dealing with many different people made it difficult for the women to build relationships with HCPs^25,28^. Relationship-based care was crucial to whether the women experienced the care as personal. Being followed by the same person and receiving continuous information and explanations in everyday language was appreciated. For some women, HCPs became ‘anchors’ amid the chaos^22,23,25^. One woman expressed the care she received as follows:

*‘No, the care up to that point had been so good, and everybody had been, um, so supportive, not that I’d expected otherwise, but I was quite, um, pleased with the time they took just to reassure me and ... that sort of personal touch, do you know what I mean, was really nice.’* ^23^

## DISCUSSION

The objective of this meta-ethnography was to explore women’s experiences of PPH to develop new insights and understanding of women’s need for care and follow-up after a PPH. We found that when the birth ended with a PPH, emotional chaos ensued. Our first theme, ‘death roams by,’ encapsulates the intense fear of death and severe pain, making the situation almost unbearable for the women. The second theme highlights the ‘emotional scar’ left by the traumatic experience. The women found it challenging to move forward without processing their experience. In this context, HCPs served as ‘anchors’ in the midst of emotional turmoil, enabling the women to emerge stronger from the experience.

The synthesis of these experiences produced the metaphor of ‘Being touched by death while giving birth to life’. This metaphor offers a new conceptual understanding and more nuanced data comprehension. Our study emphasizes the critical role that healthcare providers, especially midwives, play in offering compassionate care to help women emerge stronger from such situations.

In the following, we use Antonovsky’s theory of salutogenesis to elaborate our findings. Salutogenesis encompasses the concept of a ‘sense of coherence’ (SOC). The SOC is necessary to maintain good health and consists of three core components: comprehensibility, manageability, and meaningfulness^30^. Comprehensibility is the cognitive part and is about the extent to which a person is able to anticipate and understand a situation. Manageability is described as the behavioral part, reflecting whether the person has sufficient resources to meet the demands they are faced with. Meaningfulness is about motivation and is linked to how the person engages with and adds meaning to the situation^30,31^.

Our findings point to how postpartum hemorrhage is experienced as a situation where women are affected by fear of death. This can be understood as an existential experience. Moreover, birth, inseparable from death, can also be considered an existential experience. Existential experiences can arise as a result of an internal process or an external event^32^. We understand pregnancy, childbirth, and becoming a mother are experiences influenced by internal and external processes. ‘To be touched by death while giving birth to life’ refers to the experience of feeling that death is close at the same time as giving birth to a new life. When these two existential experiences occur at the same time, existential anxiety is amplified. Existential anxiety is a normal reaction that can strengthen or weaken a person, depending on how it is handled^32^. One study that examined women’s near-death experiences revealed that they led to emotional stress and that there was a great need for support afterwards^33^. This coincides with our findings and underlines how the experience of being close to death during or after childbirth is not a phenomenon that is only about the situation at the time. For the woman, the experience can be life-changing and have major consequences. In our study, the negative consequences of the experience are visible. Other studies point to the positive consequences^33,34^, demonstrating that the experience led to a positive change, where women could see life in a different light. They became more concerned with what was important in life and less focused on ‘trivialities’. The women felt they had been given a ‘second chance’ in life^33,34^. However, a recent study also noted that women could experience a positive change. It concluded that this was only possible when they had processed the experience and could reflect on it as a past event. The importance of support after the PPH to be able to process the experience was a key finding^35^. However, our results showed that emotional support was inadequate in retrospect. This made processing the ‘emotional scar’ a difficult process.

For many women, being able to breastfeed the child became part of processing the ‘emotional scar’^36,37^. Our findings highlight how a PPH is an event that is difficult to make sense of. The desire to breastfeed can be understood as giving meaning to the situation. Furthermore, our findings show that it was important for women to find meaning in order to move forward. Meaningfulness is not about every situation being perceived as meaningful but rather that women can use their resources to give meaning to the situation^30^. It is conceivable that women’s ability to use their resources may be affected by whether they feel strengthened or weakened by the experience. Healthcare professionals can ensure that they create a space where women can emerge more strongly from the situation by acknowledging their needs and allowing them to make decisions^38^. One study shed light on how women experienced a connection between achieving breastfeeding and being a good mother. Not being able to breastfeed intensified the feeling of failure^36^. We understand that breastfeeding is strongly linked to a woman’s maternal instinct; therefore, breastfeeding a child strongly affects the mother’s and child’s attachment. A lack of attachment makes it difficult for women to share with others, as it is linked to feelings of shame^37^. Postpartum hemorrhage makes breastfeeding less likely, and these women need extra help and support when starting breastfeeding^39^.

For the woman to feel that she is handling the situation regardless of whether she succeeds or not in breastfeeding, it is crucial that she receives support. Healthcare providers can be understood as external resources, but women also need to use their internal resources. Healthcare providers have an important role in strengthening and promoting the potential that resides in every woman. By offering physical and emotional support, the woman can gain the confidence to handle the uphill battles she faces^30,38^. Breastfeeding pressure from HCPs can amplify the experience of failure^36^. This shows the importance of being open and responsive to what is meaningful to the woman. If a person proceeds without first listening to the woman’s wishes and needs, there is a risk that well-intentioned support will instead be perceived as pressure.

Downe et al.^14^ emphasize that maternity care should be responsive to women’s values, beliefs, and needs. How maternity care enables or limits what matters to the woman is crucial for how she experiences short- and long-term births. Even when the process is unpredictable and painful, there is inherent value in being able to use one’s own physical and psychosocial capacity. How the woman is able to carry out what is important to her is influenced by expected and actual encounters with HCPs^14^. Our results confirm that how women are met by HCPs can have a major impact on their experience. Downe et al.^14^ noted that, in addition to medical security and technical expertise, women want emotional support. Recent research has confirmed that women consider safety to be something more than medical safety. Safety also means feeling emotionally and relationally cared for. Women want and believe that having personal and respectful care and continuity is important and that pregnancy, childbirth, and the postnatal period are viewed as a whole^40^. Continuity and relational security emerge in our results as important for women to experience care as personal and meaningful. A systematic review has examined women’s perspectives on the factors that increased satisfaction with continuity of care and concluded that the relationship itself is at the core. This relationship enables trust, empowerment, and care to be perceived as personal^41^. The woman’s experience of comprehensibility is greatly challenged when a PPH occurs. Even if a woman is prepared for childbirth, PPH can be an unforeseen and unpredictable experience. Healthcare providers, therefore, play an important role in helping the woman put the incident into an understandable context. By HCPs actively investing in understanding the woman as a unique person, care can be adapted individually. Our results indicate that women who experienced differentiated care to a greater extent had their needs met and experienced healthcare personnel as ‘anchors’ amid the chaos.

Although nonclinical methods such as emotional support, continuity, effective communication, and respectful care are often inexpensive to implement, in many places, these methods are not prioritized in the same way as clinical treatment is. These factors can optimize the quality of care for women^42^. By using a salutogenic perspective, healthcare providers can strengthen women’s capacity to use their resources to address challenges and move towards better health^38^. Women in labor can find themselves in all areas of the continuum between health and ill health. Having a salutogenic perspective on the midwifery profession does not mean that pathology or other risk factors are ignored^38^. However, it can be understood as a way of looking beyond the external and instead seeing the potential of the woman giving birth. By promoting women’s resources, HCPs can help women to emerge more strongly from their situation.

### Strengths and limitations

A strength of our study is that analyzing data from several studies leads to new and broader knowledge and understanding of how women experience PPH^15-17^. Our meta-ethnography included eight primary studies. A limitation of the study is that the literature search was not extensive. We searched three different databases with limited keywords, and there are likely studies that address the topic that were not identified in our searches. However, we did not exclude studies based on publication year. Although our sample included only eight studies, these contained rich and varied data material that was important for providing a deeper understanding^18^. However, the included studies are published in high-income countries, and studies describing women’s experiences with PPH in low-income countries are likely to demonstrate different results. The seven phases of Noblit and Hare^15^ were used for analysis, and eMERGe^17^ reporting guidance ensures that the analysis process is transparent to the reader. The quality of the included studies was assessed via the CASP checklist^21^. A strength of our study is that we conducted the study in collaboration. Several authors can bring different perspectives that can strengthen the study^43^. A further strength of our study is that, throughout the analysis process, we have engaged in open discussions and been aware of our preconceptions. The authors’ backgrounds had a significant influence on the analysis. All authors are authorized nurse-midwives with varying clinical experience caring for women after a postpartum hemorrhage. Further, the authors have diverse research experience, spanning various qualitative methods, including meta-ethnography. This expertise facilitated a reflexive interdisciplinary analysis and subsequent synthesis.

## CONCLUSIONS

Our findings point to how a postpartum hemorrhage is not a ‘there and then’ phenomenon but rather an existential experience that leads to an ‘emotional scar’. Our results indicate that postpartum emotional support is inadequate. Although the women survived and had a healthy child, fear of death affected them in a way that threatened their sense of coherence. To justify the experience based on survival is to downplay the woman’s emotional needs. Women need help and support to understand, handle, and find meaning in situations. To emerge more strongly from the situation, healthcare providers must recognize the woman as a ‘real’ person and provide individually tailored care.

## Supplementary Material



## Data Availability

The data supporting this research can be found in the Supplementary file.
