# Supplementary material for: Being touched by death while giving birth to life: A meta-ethnography exploring women’s experiences with postpartum hemorrhage
Source: Eur J Midwifery. 2025 Mar 26;9:10.18332/ejm/200615. doi: 10.18332/ejm/200615 (PMC11938736; doi:10.18332/ejm/200615)
Supplement: Supplementary file 1 [file EJM-9-18-s1.pdf]

Supplementary Table 1: The eMERGe meta-ethnography reporting guidance*

A meta-ethnography exploring women's experiences of postpartum haemorrhage. Eight studies published between 2011 and 2023 were included, involving 309 women from Australia, New Zealand, England, the USA, Canada and Denmark

Criteria Headings	Reporting Criteria	Pages
**Phase 1 Selecting meta-ethnography and getting started**<br>*Introduction*<br>1.Rationale and context for the meta-ethnography<br>2. Aim(s) of the meta-ethnography<br>3. Focus of the meta-ethnography<br>4. Rationale for using meta-ethnography	Describe the gap in research or knowledge to be filled by the meta-ethnography and the wider context of the meta-ethnography<br>Describe the meta-ethnography aim(s)<br>Describe the meta-ethnography review question(s) (or objectives)<br>Explain why meta-ethnography was considered the most appropriate qualitative synthesis methodology	1<br><br><br>2<br>2<br><br>2
**Phase 2 Deciding what is relevant**<br>*Methods*<br>5. Search strategy<br>6. Search processes<br>7. Selecting primary studies<br>8. Outcome of study selection	Describe the rationale for the literature search strategy<br>Describe how the literature searching was carried out and by whom<br>Describe the process of study screening and selection, and who was involved<br>Describe the results of study searches and screening	3<br>3<br><br>3<br><br>3-4
**Phase 3 Reading included studies**<br>*Methods*<br>9. Reading and data-extraction approach<br>*Findings*<br>10. Presenting characteristics of included studies	Describe the reading and data-extraction method and processes<br>Describe characteristics of the included studies	4-5<br><br>5
**Phase 4 Determining how studies are related**<br>*Methods*<br>11. Process for determining how studies are related<br>*Findings*<br>12. Outcome of relating studies	Describe the methods and processes for determining how the included studies are related:<br>-Which aspects of studies were compared<br>-How the studies were compared<br>Describe how studies relate to each other	4-5<br><br>4-5
**Phase 5 Translating studies into one another**<br>*Methods*<br>13. Process of translating studies<br>*Findings*<br>14. Outcome of translation	Describe the methods of translation:<br>-Describe steps taken to preserve the context and meaning of the relationships between concepts within and across studies<br>-Describe how the reciprocal and refutational translations were conducted<br>-Describe how potential alternative interpretations or explanations were considered in the translations<br>-Describe the interpretive findings of the translation	5<br>5
**Phase 6 Synthesising translations**<br>*Methods*<br>15. Synthesis process<br>*Findings*<br>16. Outcome of synthesis process	Describe the methods used to develop overarching concepts ('synthesised translations') and describe how potential alternative interpretations or explanations were considered in the synthesis<br>Describe the new theory, conceptual framework, model, configuration, or interpretation of data developed from the synthesis	6<br><br><br>6-12
**Phase 7 Expressing the synthesis**<br>*Discussion*<br>17. Summary of findings<br>18. Strengths, limitations and reflexivity	Summarise the main interpretive findings of the translation and synthesis, and compare them to existing literature<br>Reflect on and describe the strengths and limitations of the synthesis:	12-15

19. Recommendations and conclusions	Methodological aspects: for example, describe how the synthesis findings were influenced by the nature of the included studies and how the meta-ethnography was conducted<br>Reflexivity—for example, the impact of the research team on the synthesis findings<br>Describe the implications of the synthesis	15-16<br><br><br><br><br>16

*France EF, Cunningham M, Ring N, et al. Improving reporting of meta-ethnography: the eMERGe reporting guidance. BMC Med Res Methodol. 2019;19(1):25. doi:10.1186/s12874-018-0600-0

Supplementary Table 2: Search history

A meta-ethnography exploring women's experiences of postpartum haemorrhage. Eight studies published between 2011 and 2023 were included, involving 309 women from Australia, New Zealand, England, the USA, Canada and Denmark

Date	Database	Search terms and combinations	Limitations	Hits	Articles to consider for inclusion
14.01.2024	Medline	S1: MH "Postpartum Hemorrhage" OR "postpartum hemorrhage* OR postpartum bleeding* OR pph* OR postpartum haemorrhage*"<br><br>S2:MH "Women"OR "Women*" OR "Woman*"<br><br>S3: Experience*<br><br>S1 AND S2 AND S3	None	22 915<br><br>1 710 676<br><br>1 407 125<br><br>804	Rosvig et.al, 2023<br>Briley et.al, 2021<br>Dunning et.al, 2016<br>Elmir et.al, 2012<br>Snowdon et.al, 2012<br>Thompson et.al, 2011<br>De la Cruz et.al, 2013
15.01.2024	Cinahl	S1: MH "Postpartum Hemorrhage" OR "postpartum hemorrhage* OR postpartum bleeding* OR pph* OR postpartum haemorrhage*"<br><br>S2:MH "Women"OR "Women*" OR "Woman*"<br><br>S3: Experience*<br><br>S1 AND S2 AND S3	None	4215<br><br>541 319<br><br>575 882<br><br>242	Snowdon et.al, 2012<br>Dunning et.al, 2016<br>Elmir et.al, 2014<br>Elmir et.al, 2012<br>Thompson et.al, 2011<br>Briley et al. 2021

15.01. 2024	Maternity and Infant Care (MIDIRS)	S1: Postpartum hemorrhage* OR postpartum bleeding* OR pph* OR postpartum haemorrhage*<br><br>S2: Women* OR Woman*<br><br>S3: Experience*<br><br>S1 AND S2 AND S3	None	4451<br><br><br>129 001<br><br>39 328<br><br>402	Thompson et. al, 2011<br>Rosvig et. al, 2023<br>Briley et. al, 2021<br>Dunning et. al, 2016<br>Snowdon et. al, 2012<br>Elmir et. al, 2012
**Comments:**<br><br>Siteringssøk med artikkelen Dunning et. al, 2016.<br>Antall treff: 67. Ingen artikler valgt ut.<br><br>Siteringssøk med artikkelen Snowdon et. al, 2012.<br>Antall treff: 60, ingen nye artikler valgt ut<br><br>Siteringssøk med artikkelen Briley et. al, 2021.<br>Antall treff: 14, ingen nye artikler valgt ut<br><br>Siteringssøk med artikkelen Rosvig et. al, 2023.<br>Antall treff: 1, ingen nye artikler valgt.<br><br>Siteringssøk med artikkelen Elmir et. al, 2012.<br>Antall treff: 116, ingen nye artikler valgt ut.<br><br>Siteringssøk med artikkelen Thompson et. al, 2011.<br>Antall treff: 44. Artikler valgt ut: Robertson et. al, 2017.<br><br>Søk i referanselister på alle utvalgte artikler. Ingen relevante artikler valgt ut.					

Supplementary Table 3: PICO-form

A meta-ethnography exploring women's experiences of postpartum haemorrhage. Eight studies published between 2011 and 2023 were included, involving 309 women from Australia, New Zealand, England, the USA, Canada and Denmark

**Population**	**Intervention**	**CO**<br>**Comparison**<br>**Outcome**
Women<br>Women*	Experience	Postpartum hemorrhage<br>Postpartum hemorrhage*
Woman*		Pph*
		Postpartum haemorrhage*
		Postpartum bleeding*

Supplementary Table 4: Literature matrix

A meta-ethnography exploring women's experiences of postpartum haemorrhage. Eight studies published between 2011 and 2023 were included, involving 309 women from Australia, New Zealand, England, the USA, Canada and Denmark

Rosvig et.al (2023)	Dunning et. al (2016)	de la Cruz et. al (2013)	Elmir et.al (2012)	Snowdon et. al (2012)	Briley et. al (2021)	Robertson et. al (2017)	Thompson et. al (2011)	Our translation
1) The seriousness of the situation became more visible when more health personnel were called, alarms were activated, and the women noticed a tense atmosphere among the health personnel<br><br>*"I could feel the blood pouring out of me. And they called more people in and started to wheel me out... And I panicked. I thought: '****, Am I going to die?' I mean, that*		1) Most of the women were not aware that the severity of the situation, and that death was actually a possibility. Some women reported feeling calm and not scared during the bleeding itself.<br><br>2) A few women felt like they were dying<br><br>*... I'm going in and out of consciousness, and I'm having a hard time staying earth-bound...I'm just talking to my husband and telling him, "Please make sure that you take care of our kids and*	1) The women experienced fear and uncertainty about whether they would survive due to the large blood loss. The feeling of fear and shock led to chaos of thoughts.<br><br>2) The women described the postpartum haemorrhage as a situation where they were between life and death.<br><br>3) The women feared for the	1) The women had a need to move on and focus on the child. They would not think about the consequences of postpartum haemorrhage, and especially death, would have affected their family. The women pushed their thoughts away and did not want to deal with death.			1) Some women experienced symptoms of post-traumatic stress disorder after the incident.<br><br>*"I think that as a result of what felt like a ''brush with death,'' I am more anxious about losing my partner or baby. They are more precious than ever"*	«A brush with death»<br><br>Death knocks on the door? Death sweeps by?<br><br>1. Being afraid of dying<br>2. To be confident that you will not die<br>3. Not wanting to think the thought<br><br>Existential worries, existential crisis<br><br>Acceptance is hard, emotional scar Fera

was my first thought"<br><br>2) Most of the women experienced the situation as potentially life-threatening, but quickly dismissed the thought as irrelevant.<br><br>3) Some women experienced a strong fear of death. They could "feel" that they were losing a lot of blood, and experienced that health personnel worked quickly around them.		*let him [newborn] know that I loved him very much if I don't make it through all of this." I remember kind of losing it before I went into surgery, almost like leaving earth, like having an out-of-body experience.*	lives and future of their children, partners and other members of the family.<br><br>*'d lost so much blood so quickly, I was dying, basically, and I could feel myself dying and sort of slipping away which. I felt like, you know those really old TVs, where you turn them off and the screen sort of disappears into a little circle before it goes black*<br><br>4) The women felt that they were the "backbone" of the family and feared for their children's					

			future, and how they would manage without a mother.<br><br>5) Some women sought security in their religion and faith during the acute situation when they feared for their lives.<br><br>6) *The nightmares usually revolve around the part where I am begging for help, it's really horrible. Because the pain is unbearable, I have nightmares where I am trying to see people's*					

			faces, cause I remember gasping for breathy In the nightmare, I don't remember pain, I always remember I am about to die. So the nightmares are about me about to die after giving birth					
1) The women experienced the pain as intense, and shouted and screamed. The women stated that the pain compared to the birth pain was easier to bear, as they expected the pain to last shorter. None of the women stated that they were not well relieved of their pain.		1) Consistently tell the women about severe pain related to the bleeding. The women who gave birth vaginally seemed to have stronger pain during the bleeding itself, compared to the birth pains<br><br>2) The intensive care stay involved severe pain regardless of the delivery method.		*I just remember lots of people pummelling at my stomach in shifts, like two people at a time, pummelling my stomach. And I can remember it hurting and just actually trying to push them away.*		1) The pain during the bleeding was experienced a million times worse than the birth itself. Especially the pain associated with bimanual uterine compression, manual removal of the placenta and squeezing out clots.		The pain was experienced as equal to or worse than the birth pains<br><br>Memories of shouting and screaming<br><br>A desire to be disconnected

		...she [obstetrician] started pounding on me with her fists. It was such horrible pain that I couldn't think of anything other than the pain, and then when she said probably 5 or 10 minutes later, "We're going to take you into surgery." The only thought was, "I just want to be put out. I'm so looking forward to going into surgery and to be put under anesthesia... so that I can't feel the pain anymore."<br><br>3) Some women experienced the severe pain as traumatizing<br><br>*...I said the epidural hadn't worked, but she [obstetrician] didn't believe me, I guess, even though the anesthesiologist said it didn't work. And so she reached in and she manually*				*The nightmares usually revolve around the part where I am begging for help, it's really horrible. Because the pain is unbearable, I have nightmares where I am trying to see people's faces, cause I remember gasping for breathy In the nightmare, I don't remember pain, I always remember I am about to die. So the nightmares are about me about to die after giving*		

		tried to remove my placenta. Which is the most painful thing in the world to have happen, and to have no pain relief. It was very traumatic. Later, I couldn't close my eyes because I would have visions of her doing that to me, even though I hadn't seen it...I do not have fond feelings for this doctor						
1) The women who were allowed to have their child with them felt that the child acted as "a filter" against the chaos around them. They had all their focus on their child.<br><br>2) Some women described how they completely dissociated themselves from the whole	1) The women lost control of the time during the acute incident, and the treatment afterwards.<br><br>2) Dissociation was a consistent finding among the women, regardless of the amount of bleeding<br><br>*'I was so out of it that ... I didn't*		1) The women felt helpless and weak, and described an "out-of-body" experience, and that they felt disconnected from their own bodies.<br><br>*I actually at one stage can remember detaching so much that I felt like I was*					A distinction between body and consciousness<br><br>To "disconnect" from one's own body<br><br>Seeing yourself from the outside<br><br>Traumatic experience

situation, and instead focused on staying calm and dealing with the pain.<br><br>*"I just think I surrendered myself completely to the situation. Like, I am completely powerless, and I have absolutely no clue what's going on. So, I was just like... 'do whatever you want with me', you know. Because you are... completely surrendered to the situation and I was ready for... whatever works, right "*	*really think, I didn't even know what was going on really'–W7 (Severe)*		*looking at myself on the table with everyone around me that really profoundly affects me inside (Jade).*<br><br>2) *I had a major anxiety attack I think I made the connection between feeling really cold [due] to the blood loss and the shock that I was going into, and the fact, you know, how close to death people can be in those times. I felt like I was kind of having a bit of an out of body experience. I was disassociating from it*					

1) Transfer to an operating theatre was described as the greatest fear for the women, and several women said that they were willing to endure severe pain and manual procedures in the delivery room rather than being transferred to an operating theatre.<br><br>2) Information about the next plan/next step led to the women feeling safe because the women gained confidence that the health personnel were in control of the situation. It was reassuring for the women to see that health personnel normalised the situation. The women observed that healthcare	1) Some women felt like they were losing control.<br><br>*'I felt like I had no control and that they didn't know how I was feeling and ... whether they were going to be able to deal with the issue. Obviously I knew that they could because they were a hospital, but in that second in that situation all sorts runs through your head'*	1) Many women told of fear during pph and during the hospital stay afterwards.<br><br>2) The feeling of fear became prominent when separating the child, when the partner was escorted from the room, or when the health personnel called for blood products.<br><br>3) The fear was related to not knowing what had happened, being alone or threatening one's physical integrity. The way the health personnel behaved had a major impact on the women.		1) The women described the experience as traumatic and frightening.<br><br>*[N]urses were just coming in, rushing in from God knows where, I mean I don't know how many there was and it felt like no one was telling me what was going on. I mean I was just lying there thinking 'Oh God, oh God, what's happening?' I suppose 'cos they were so concerned that I was bleeding so much. [T]hey were putting like stuff in me hands and y because*		1) The women felt that they were involved in the process, and decisions were made with their consent. The women greatly appreciated this.	1) The women expressed concern about whether the care/help they received was good enough. Both during the hospital stay, but also afterwards. They were particularly concerned about whether ppb could have been avoided, and whether the information they received was sufficient.	The experience of losing control<br><br>The importance of getting involved in decisions, feeling in control<br><br>Relinquishing control felt safe for some, others felt they had to be involved in the process to be safe.<br><br>Trust/distrust of healthcare personnel<br><br>Body language among healthcare personnel led to distrust/trust

personnel were in control and knew what was going to happen next, and informed the women continuously.<br><br>3) A few women felt vulnerable during the transfer when they encountered other healthcare personnel or visitors during the transfer. The operating theatre was described as a stark contrast to the delivery room. The operating theatre was described as a cold, bright room with even more strangers.				*they wasn't talking to me, I was worried, I was panicking.*<br><br>2) The women described themselves as passive participants, and did not expect to be involved in the decisions that were made. It was out of their control.				
1) As the physical fitness of the women gradually improved, the shock of ppb subsided. Several of the women explained how	1) The women who had had a severe postpartum haemorrhage tried to understand/make sense of ppb	1) A few women experienced fear during the follow-up consultations. Everything from being touched and examined, and the fear of reliving the	1) The emotional scar from the experience was a concern for the women to a greater extent	1) The inability to obtain information, and protest in the event of a lack of information,		1) Anxiety, nightmares and flashbacks were described by some women. These feelings lasted longer than the	1) Many women described that they had not received sufficient information to be able to understand	Major emotional and physical consequences<br><br>Poor physical fitness, difficulty

they decided to move on. All the women expressed relief and gratitude that it had gone well.<br><br>2) A few women wanted a follow-up consultation to process the experience.<br><br>3) A few women felt hesitant/doubtful in relation to future pregnancy and childbirth in relation to the experience with ppb.	when they returned home.<br><br>2) Some women searched for information when they returned home, for example through the internet to try to make sense of what had happened.<br><br>*'So it wasn't until I was at home, it may have been a week or two afterwards that I Googled what it was, that I realised, and I hope my understanding is correct, I think at the time I thought it was something to do with the tear and the stitches, and then I realised it was actually more to do with the placenta, and it ... So I was ... until I Googled it*	memories of the bleeding itself.<br><br>2) Many women expressed that the health personnel did not acknowledge their emotional needs. They expressed that there was a need to devote more resources to their mental health before returning home, for example a referral to a psychologist who can talk about traumatic birth experiences.<br><br>3) After discharge, most of the women described an extreme need to fill in gaps in their memory. They asked their partner about the incident. Several women read their own medical records and dug into them. At the follow-up later, they wanted the gynaecologist's	than their physical health.<br><br>2) In addition to flashbacks, several women also experienced nightmares, which led to poor sleep quality, insomnia and difficulty sleeping. This affected women's level of functioning during the day.<br><br>3) Returning to the hospital gave the women painful memories of ppb. Smells, the sounds of healthcare personnel, and the sight of other patients triggered flasbacks,	had consequences over a long period of time. The women felt like a victim.<br><br>*[It] has affected the way that I think about myself, the way I conduct myself, my life everything, because I've never got over that anger with myself for putting up with what I put up with there. I was just a complete victim*<br><br>2) *I was never debriefed properly. I don't know what happened*		postpartum period, even into future pregnancies.<br><br>*«I think I experienced not severe posttraumatic stress but something like that. I would have flashbacks to the pain, mostly the pushing on my stomach. I can still feel that. It hurt and it was a bit traumatic, and I didn't recognize it as trauma…I think I just thought it was normal to have those feeling after birth…But I think…I just didn't realize that it was traumatic. So I'm still*	what had happened to them during childbirth and PPB.<br><br>2) The women particularly expressed a need for information about the course of events and what potentially caused ppb.<br><br>3) The women described feelings such as disappointment, bad thoughts and bad memories.<br><br>4) More women expressed that they would like more information about what they could expect after a PPB, and what kind of support was available.	performing everyday activities<br><br>Need for information in retrospect to understand<br><br>The emotional "scar"<br><br>Need for more emotional support afterwards<br><br>Desire to be recognized<br><br>Flashbacks, bad memories, triggers<br><br>Filling in "gaps" in the memories<br><br>Choice not to have any more children

	myself I was completely unaware of what had actually happened.'<br><br>3) For most of the women, it took a long time to recover physically regardless of blood loss. The women talked about using iron tablets for months after giving birth, and they found everyday activities such as walking difficult.<br><br>4) Most of the women had recovered mentally and emotionally in the time after ppb.<br><br>5) The women expressed that they wish they had received more information during the postnatal period.	perspective on what had happened.<br><br>4) Although some women found it stressful to be reminded of the trauma/event, and avoided the hospital, it was helpful for others to return to the hospital where everything had happened in order to process the memories.<br><br>5) The women wanted information in all phases of the process. Health professionals who were able to fill in these gaps in their memories helped the women accept what had happened so that they got a closure and was able to move on.	which led some women to hyperventilate and become anxious.<br><br>4) Some women described a fear of not being in control in everyday life, and this anxiety was amplified when they were in situations where they were not in control of their own body.<br><br>*I still get very anxious. Ever since it happened I've hated being a passenger in a car and even now, I don't know if it's the loss of being out of*	*during them days. It was all coping with the trauma and coping with the new baby yit probably took me till about six to eight months to actually come up with some of these questions that I wanted answers to, that Jerry couldn't answer 'cos obviously he didn't know the technicalities of it. So I feel like I've been left quite ignorant y. To this day I don't know what's happened.*<br><br>3) A woman described that she was discharged		*dealing with it, I think»*<br><br>2) The women discussed the benefits of being able to lean on supportive networks, and focus on their own children to help them recover.<br><br>3) Some felt it helped to write about their experiences.<br><br>4) Many women described that recovering physically was tiring, tiring and took longer than expected.<br><br>*«I remember the extreme exhaustion. I remember trying to wash my hair and I*	5) Some women talked about slowly but surely coming to terms with what had happened to them.<br><br>6) Some women expressed a desire for access to more support from healthcare professionals after discharge, to help them cope with the emotional chaos.<br><br>7) Some women described that they had come to terms with what had happened and accepted the negative memories.<br><br>8) Several women said it would have been helpful if they had	

			control but I can barely be a passenger in a car. I always have to be the person driving and I was never like that before and I just have this fear of car accidents	from the hospital prematurely, and she thought it would have been useful for her to talk to someone about what had happened before she went home.		*couldn't lift my hands up, I just felt so faint and tired»*	received more information about how they could expect to feel after ppb.<br><br>9) The women worried about consequences related to ppb, such as whether it would affect fertility, fear that it would happen in a future pregnancy. They also talked about the loss of faith that they would be able to safely give birth to a new child. For some women, it led to a choice not to have any more children.<br><br>10) The women described fear of a new ppb or a new traumatic birth in future pregnancies.	

							"I must admit that the thought of going through labour again scares the wits out of me. I almost feel like I cheated death once and don't want to tempt fate"<br><br>11) Some women described deficiencies in postnatal care and follow-up.<br><br>*"I do sometimes wonder how it is possible that I was allowed to go home two days after the birth and postpartum hemorrhage"*<br><br>12) The women suggested that further information about what they could expect in	

							relation to physical fitness after ppb would have been helpful. They were interested to know how long it would take for them to get back in shape, and if they would need additional support.<br><br>*“I was not given information regarding the extra time recovery would take after losing a lot of blood. I felt I was not coping as I compared myself to women who had more ‘‘normal’’ births without blood loss”*<br><br>13) *“I still think about my haemorrhage a lot; it was a very scary time and*	

							not something you prepare for when you have a baby. Most people in my life, however, have forgotten already how traumatic it was, and that leaves me feeling very lonely when thinking about what happened"	
1) The women appreciated the early attachment to the child, some felt that the postpartum haemorrhage prevented them from having this opportunity<br><br>2) The women appreciated that the health personnel made arrangements for the woman, partner and child to be gathered at	1) Separasjon was a major concern for all the women in the study. The women spoke of a sadness because of the separation from their child.<br><br>2) A woman told of challenges related to breastfeeding, which resulted in the child being rehospitalized. The woman was	1) The women who had poor health after ppb experienced a prolonged separation from their child. This led to a feeling of sadness, guilt, and regret. Some women described an urgent need to be with their newborn child.<br><br>*I was really worried because I'd not seen him [newborn] for so long—I'd not*				1) The separation from the newborn child during the management of the postpartum haemorrhage was described as difficult and stressful.<br><br>2) Most of the women stated that they did not have problems with breastfeeding.	1) Separation from the child, attachment and delayed onset of breastfeeding were a concern for the women.<br><br>*"I feel the 'bond' with my son weakened significantly due to my postpartum recovery "*<br><br>2) Several women said that it bothered	Feelings of loss and disappointment over the loss of the perfect birth, and lost time with the newborn baby<br><br>Guilt over not being there for your child, being inadequate as a mother<br><br>A desire to breastfeed

the "head of the woman"<br><br>3) The women were painfully aware that the transfer to the operating theatre would lead to separation from the child<br><br>3) The women told how their first meeting with the child was postponed until they woke up. For many women, this moment was not so magical as they felt dizzy and extremely exhausted.<br><br>4) During the days in the maternity ward, the women were still exhausted and dependent on help from their partner for everything practical, such as changing diapers,	then told several weeks later that this could be related to her PPB.	*even held him—that I'd struggle to bond with him and I wouldn't be able to feed him, and not only would I not be able to have the delivery I wanted, I also wouldn't be able to feed him myself... I just couldn't wait to hold him, almost frantic... I was worried and I feared that I wouldn't be able to bond with him very well*<br><br>2) Some women were physically exhausted and not immediately worried about their child, resulting in feelings of guilt later on.<br><br>3) All the women had a desire to breastfeed, and some felt that they did not receive sufficient support for it.				 3) One woman was satisfied with not receiving information about how ppb may have affected milk production.<br><br>4) Some women who experienced challenges related to breastfeeding believed that this could be related to separation from the child, fatigue and lack of support from healthcare professionals.<br><br>*«My lack of strength, energy, and pain during recovery made breastfeeding effectively a major challenge»*	them that they did not feel strong enough to take care of their child.	Challenging to breastfeed<br><br>Breastfeeding as "healing"

helping with breastfeeding and helping the woman to the bathroom. This led to some of the women feeling inadequate and guilty for not being 100% present for the child and partner, which led some women to see the days after ppb as just as horrible as the ppb itself.<br><br>*"The first two weeks, I felt so inadequate as a mother because I was so weakened. It took me a long time to hold her, because I did not trust my own strength. And (partner) took care of everything in the beginning – feeding her, changing diapers, all that stuff. (...)*		4) A few women succeed in breastfeeding fully, which for them was positive and healing.<br><br>5) Most supplemented with infant formula and felt that getting to breastfeed at all was an achievement.<br><br>*In the maternity ward the one thing that really, really bothered me the most was that they didn't give me enough information about breastfeeding and they kind of tried to steer me away from breastfeeding, "Oh you shouldn't bother breastfeeding you've gone through so much trauma. It's not going to work and blah blah blah." I just wish they would have just listened to*				*"Emotionally, the guilt of not being there for my child immediately after birth was difficult."*		

That's not how it was supposed to be."		*my wishes and helped me out a little bit more. And I know they thought it was bad for me, but it wasn't, emotionally anyway.*<br><br>6) Most of the women wanted to have the child with them in the same room. An arrangement that was emotionally necessary, despite challenges with physical fitness.						
1) The women felt safe in the acute situation and were impressed by how quickly and efficiently the team worked<br><br>2) Some women talked about the fact that consent was not necessary in the handling of PPB. Some expressed that they	1) Women with severe ppb were generally satisfied with the handling of the situation, and felt seen and safe. The healthcare personnel were perceived as competent and safe<br><br>*'No, the care up to that point had been so good and everybody had*	1) The women expressed a special need for care characterised by compassion, respect and sensitivity.<br><br>2) The women felt that they were deprived of the "perfect birth". They experienced a loss, a grief because of this. The women who underwent hysterectomy		1) Some women experienced that the healthcare personnel withheld information, and this led to them becoming suspicious and vulnerable.<br><br>2) The women were	1) Although the incident was perceived as frightening, the women felt that the treatment was quick and effective and they felt that the situation was handled well and efficiently.<br><br>2) The women talked about		1) The women were worried about whether the help they received was good enough, and whether the situation could have been avoided.<br><br>2) Some expressed a lack of trust in the health personnel.	Health professionals Care and communication<br><br>From woman giving birth to patient<br><br>Health personnel as an "anchor" in the chaos<br><br>Personal touch Personalized,

appreciated that professional health personnel were in control of the situation.<br><br>3) The women described a change in their role from woman giving birth to patient. This led to a change in communication between healthcare personnel, which made even more clear the seriousness of the situation. The women described it as a "turning point". No way back.<br><br>*“The OR really upset me. Like, I felt like everything was hanging out of me and... I felt so powerless. They moved me around, like: ‘I am just going to lift your arm’*	*been, um, so supportive, not that I’d expected otherwise but I was quite, um, pleased with the time they took just to reassure me and ... that sort of personal touch, do you know what I mean, was really nice’*<br><br>2) Most of the women experienced a sense of control during the incident itself, this related the women to the care they received from the health personnel.<br><br>3) The women described that information could also cause more fear and insecurity if it was given at the wrong time.	experienced a loss of fertility and femininity. The women wanted healthcare personnel to acknowledge this feeling of grief.<br><br>3) Communication was a key issue, not only in terms of what kind of information they received, but also how and when they received the information.<br><br>4) The women felt calmer during ppb if the health personnel were able to reassure<br><br>*She [obstetrician] was just so good at getting in my face and telling me what was going on. She was very calm, but very matter-of-fact, and I never worried about it... I think that that was probably the most helpful, her attitude*		willing to forgive and move on if they were given the information they were promised. If it was not followed up, it became difficult to move on and get rid of the "anger".<br><br>3) Although the women experienced pain and were confused when they woke up after the anaesthetic, they experienced a strong and immediate urge for information	how they saw through healthcare personnel who tried to act calmly, by observing their facial expressions.<br><br>*“ . . . I just had a baby . . . and she [Midwife] was very nervous, I remember thinking that wasn’t a very nice face . . . ”*<br><br>3) Although verbal reassurance was helpful, the body language of the healthcare personnel was revealing of the severity of the situation.<br><br>4) The women were aware that it is difficult to estimate		*“I did not know the midwife before the delivery, and I found her to be very unsupportive and impatient towards me. When I began haemorrhaging shortly after delivery, she made a great effort not to acknowledge me as she tried to stop the bleeding. But as she failed to hide her panic and she had never once assured me that I could trust her, I was terrified!!”*<br><br>2) Some women noted that they felt that there was poor staffing.	personalized care<br><br>Need for care<br><br>Health personnel who showed emotion and became emotional made the women feel like a 'real person' to them.<br><br>Body language and facial expressions revealed by the healthcare personnel<br><br>A desire for continuity

which just makes you feel even more sick. And I was so cold. And they put on suits and masks and asked me for my social security number, but I almost could not speak. It was just so overwhelming that I had suddenly ended up in that situation"<br><br>4) The women were uncertain about the seriousness of the situation, and became very aware of the communication between health personnel in order to pick up information. However, the women understood that this communication was necessary, but the medical terminology used	4) The women were not aware that they had lost more blood than usual, some women did not find out about this until they were contacted to participate in the study. This was not necessarily something the women believed would have affected how they viewed the situation.	*through it all. She wasn't freaking out, "Camilla, you need to stay awake." "Camilla, you are losing blood." "Your uterus isn't contracting down...you are bleeding more than usual."... It didn't register just how serious it was... which was good for me because I didn't really panic, ever.*<br><br>3) The women experienced a strong need for information during their hospital stay. They wanted information about what had happened and what was going to happen. Lack of information felt traumatizing<br><br>4) Lack of continuity and a person to relate to was problematic.<br><br>5) The women spoke positively			blood loss, and perceived that the healthcare personnel were unclear in relation to how much blood they had lost.<br><br>5) The women talked about how the risks of ppb should be communicated before, during or quickly after ppb.<br>6) When decisions were made about ppb, the women felt that the information about the risks was provided in a way that was informative, but did not lead to panic.<br><br>7) The women expressed that			

made the women more insecure in the situation.<br><br>5) The women overheard words such as 'anaesthetist', 'operating theatre', 'blood transfusion', and this triggered thoughts that the situation was extremely serious.<br><br>6) Continuous information and explanation in everyday language by the same person was appreciated by the women. This person became like an "anchor" in the chaos for the women.		about health personnel who showed emotions. They found this to be supportive and meaningful.<br><br>*You always have to be professional but...it's OK to let your barriers down as a health care provider, it's OK to say, "Oh my God, we were so scared, ""Oh my God, that was so awful...let's talk about it together." That was a wonderful thing. That was my experience, which was very positive... That was a very powerful thing, to feel like I was a real person to those people, and that they care too and that they hurt too. [Crying]*			it was a shock to them to hear how much blood loss they had had. They commented on how healthcare personnel appeared less worried, even though the women perceived it as a major blood loss even though the blood loss was 'only' 500 ml.<br><br>8) Some women stated that getting information about ppb immediately afterwards was inappropriate and that they were unable to understand the information at the time.			

Supplementary Table 5: Translation process

A meta-ethnography exploring women's experiences of postpartum haemorrhage. Eight studies published between 2011 and 2023 were included, involving 309 women from Australia, New Zealand, England, the USA, Canada and Denmark

Translations	Subthemes	Themes	Overarching metaphor
Several women were afraid of dying<br>Fear of leaving the family behind<br>The feeling of dying, - physical symptoms<br>Seriousness sinks in when the alarm goes off, more HCPs are called, communication in the room changes<br>Some did not understand the seriousness of the situation<br>Pushing the serious thoughts away	The fear of dying	When death roams by	“Being touched by death while giving birth to life”
Pain described as stronger than the birth pains<br>Memories of shouting and screaming, pushing hands away<br>Manual procedures in the delivery room that led to excruciating pain<br>Flashbacks to the pain<br>A desire to be "disconnected"	Stronger pain than birth pain		
Disconnecting from your own body<br>Feeling helpless and weak<br>Lost perspective of time<br>Seeing yourself from the outside, a traumatic experience<br>The child as a "filter" in the chaos<br>Being disconnected	Disconnecting from the body to endure an unbearable situation		
Being confident in HCPs, being able to relinquish control<br>Acknowledging emotional needs<br>A desire for respect, compassion, and sensitivity<br>Lack of trust, see through body language and facial expressions<br>A desire for genuine and personal care, a powerful experience that made an impression	The importance of being recognised as a “real” person	Healthcare providers being “anchors” amid the chaos	

A great need for information<br>A desire to understand what had happened and what was going to happen<br>The importance of information being provided at the right time, individually adapted<br>Ongoing information<br>Lack of continuity, difficult to build relationships	A desire to understand		
Took a long time to recover physically<br>Feelings of loss, disappointment<br>Comparison with healthy postnatal women<br>Difficult to perform everyday things<br>Painful memories, nightmares and fear for a long time afterwards<br>Hesitant to have a new pregnancy, or a decision not to have any more children<br>Feelings of loss and guilt due to separation of the newborn child<br>The feeling of being inadequate as a mother<br>Breastfeeding is chellenging<br>A desire to be able to breastfeed	Feelings of loss, disappointment and guilt over an experience that did not turn out as it should have	Living on with an "emotional scar"	
PPH affected them emotionally, worried about the emotional "scar"<br>Need to process the experience and fill in gaps in the memories<br>Perception of lack of follow-up<br>Need for information, trying to find the information yourself via the internet, partner or hospital records<br>Awareness of what they expected and needed in terms of information<br>Being unable to protest when experiencing lack of follow-up/information<br>Triggers that led to the reliving of the event	Finding meaning to be able to move forwards		

© 2025 Allum K.E. et al.
